# Effect of Different Modified Atmosphere Packaging on the Quality of Mulberry Fruit (*Morus alba* L. cv *Kokuso 21*)

**DOI:** 10.1155/2021/8844502

**Published:** 2021-02-03

**Authors:** Ilenia Tinebra, Giuseppe Sortino, Paolo Inglese, Silvia Fretto, Vittorio Farina

**Affiliations:** Department of Agricultural, Food and Forestry Sciences (SAAF), University of Palermo, Viale delle Scienze, Edificio 4, ingresso H, 90128 Palermo, Italy

## Abstract

The control of temperature and gas composition is essential to maintain the fresh flavor and quality of perishable fruits like mulberry. This study presented a modified atmosphere experiment (MAP) for fresh fruit showing the potential benefits of innovative gas mixing with argon. The effects of MAP were studied on the physicochemical and qualitative attributes of mulberry preserved at 4 ± 1°C and 90 ± 5% R.H. Fresh mulberries were packaged with different gas combinations: MAP1 (4%O_2_+6%CO_2_+90%N_2_), MAP2 (10%O_2_+5%CO_2_+85%Ar), CTR1 (20.9%O_2_+0.04%CO_2_), and CTR2 (10%O_2_+5%CO_2_+85%N_2_). Changes in quality parameters were evaluated after 0, 4, 8, and 12 days of storage. Mulberries packaged with MAP had a lower weight loss than CTR samples which lost more than 80% of their initial weight. Furthermore, the results showed that the argon treatment was the best in keeping the fruit juice content, preserving its structure. Despite not showing great differences with MAP1 treatment, Ar allowed to maintain high TSS up to 8 storage days, slowed CO_2_ production. The sensory profile of mulberry fruit was not significantly affected by storage in modified atmospheres, and the production of potential unpleasant odors in MAP2 could not be perceived. The results of this study confirm that this innovative approach, using MAP technology, has a potential use in maintaining mulberry fruit quality for a longer time.

## 1. Introduction

Within the Italian sector of fruit production, small fruits are a niche produce. The consumption of blackberries, blueberries, mulberries, strawberries, and raspberries has increased during the last 10 years, due to the gradual raise of consumers' awareness of the high nutritional value of all types of small fruit [1] and of the related benefits for human health of their consumption, because of their bioactive compounds [2]. Small fruit are characterized by a high berry perishability, rapid quality decay, limited shelf life, even if stored under refrigerated conditions. This may cause high production costs, limitations for marketability and a consequent loss of commercial value [2].

Mulberry is a deciduous woody tree belonging to the genus *Morus* of the family *Moraceae* [3], globally distributed under varied climatic conditions ranging from tropical climate to the temperate one [4]. There are about 68 species of genus *Morus* worldwide [5], but only white mulberry (*Morus alba* L.) and black mulberry (*Morus nigra* L.) are cultivated in Italy [6, 7]. Mulberry is a climacteric fruit; it is rich in carotene; vitamins B1, B2, and C; glucose; sucrose; tartaric acid; and succinic acid [8, 9]; it also contains antioxidants, total phenolic, and anthocyans [10]. Moreover, due to its particular nutraceutical value, mulberry is considered as a functional food [10, 11]. Because of these reasons, mulberries are considered a high-end product. The cost in Italy and neighboring countries ranged from a minimum of 7 euros per kg of fresh product to a maximum of 13 euros due to the high costs of production, harvesting, storage, and distribution [12]. In fact, mulberry fruit is one of the most fragile and perishable fruits, both in harvesting and postharvest, having a very short shelf life of 2–3 days, which can be greatly reduced by storage temperatures above 0°C. The color of mulberry fruit is related to the species: *Morus alba* L. has white and black fruit; instead, *Morus nigra* L. produces only black fruits. The mulberry fruit ripening is usually correlated to a skin color change due to an accumulation of anthocyans that implies a modification of pigments' concentration in surface tissues [13] and a degradation of chlorophylls and carotenoids with a consequent development of color from green to purple [14, 15].

Although, official statistics demonstrate that Italy imports small fruit from other countries, mulberry fruit is still poorly commercialized in Italy, compared to other countries, despite the increasing interest of consumers in this produce. Indeed, mechanical damage and microbial spoilage hamper their transport from the production to the processing/selling site [16, 17]. Therefore, there is much interest of stakeholders in developing preservation methods for all types of small fruit and particularly for the mulberry fruit which appears very interesting as a source of vitamins, minerals, and fiber in the human diet and for its use as a superfood or in the pharmaceutical industry. Different postharvest technologies, such as active packaging [18], edible coatings [19], oxidizing sanitation technologies [20], and modified atmosphere packaging and gaseous ozone prepackaging treatment [16, 21, 22], were proposed to preserve the quality of small fruit and extend their shelf-life. Chen et al. [23] investigated the effect of immersion in chlorine dioxide solutions (20-80 mg L^−1^) on the shelf-life of mulberry fruits and observed a shelf-life extension of up to 14 days. Teng et al. [24] observed that chlorine dioxide and electrolyzed water solutions were effective in reducing microorganisms and extending the shelf-life of mulberries. Oz and Ulukanli [25] studied the effects of 1-methylcyclopropene (1-MCP) and calcium chloride on the postharvest quality and shelf life of mulberries. Hu et al. [26] treated the mulberry fruit with a hydrogen sulphide release compound (H_2_S) and observed that the treatment increased the activity of some antioxidant enzymes and consequently reduced the production of superoxide anions in the fruit. Treatments of allyl isothiocyanate [27] and chitosan-caffeic acid [28] were found to have significant effects on the postharvest quality of the mulberry, and both treatments were proposed as potential methods to extend the shelf life of the fruit. More recently, Pinto et al. [16] and Tabakoglu and Karaca [29] reported that mulberry fruits subjected to a combined treatment with ozone had a lower rate of decay, respiratory intensity, and polyphenol-oxidase activity than control samples.

Modified atmosphere packaging (MAP) treatment effect on fruit quality was tested in strawberries, sweet cherries [30], pomegranate [31, 32], litchi [33], table grapes [34], kiwi [35], and blueberry [36]. MAP resulted in an effective method of fruit quality preservation to extend the shelf life of small fruits, with positive effects on physical-chemical parameters and a reduction in the development of fungi. For example, blueberries stored in MAP at 3°C showed a shelf-life extension of 9-15 days compared to air storage, depending on the packaging material [37], while for strawberries after storage at different temperatures [38], a shelf-life gain of more than 1 day was expected. Microbial growth control is achieved as a result of high CO_2_ concentrations [39]; however, achieving a partial CO_2_ pressure higher than 5 kPa in MAP could lead to the development of undesirable flavors in strawberries and raspberries [40] and/or may reduce the consistency of different types of small fruits [41].

The three traditional gases for modified atmosphere packaging are oxygen, carbon dioxide, and nitrogen [42, 43]. Recently, there was a great interest of researchers in the potential benefits of argon (Ar) and other noble gases in MAP applications [44, 45]. Moreover, argon was recently allowed to be used for MAP in the European Union as an alternative to nitrogen [46, 47], because it is inert, odorless, and tasteless [48]. Although inert, argon appeared to develop biochemical activities such as interference with oxygen receptor sites of enzymes and with protein conformation change. Furthermore, argon displaces oxygen more effectively than nitrogen. This is possibly based on its atomic size which is similar to molecular oxygen, its higher water solubility (0.034 vs. 0.016 g·L^−1^), and its density which is higher than that of nitrogen (i.e., argon 1.650 kg/m^3^ vs. nitrogen 1.153 kg/m^3^) [49, 50]. With regard to the inhibitory activity against bacterial growth, argon was deemed to have a better solubility in fat, which results in improvement of membrane permeability of CO_2_, salts, and acids to bacterial cells [51].

Several studies were conducted to investigate the effect of argon on enzyme activities and sensory characteristics of fruits and vegetables [52–56]. Various authors reported the effectiveness of Ar in limiting the growth of certain microorganisms, suppressing enzymatic activities, and controlling degradative chemical reactions in several perishable food products, such as mulberry fruits, which have been reported to show microbial activity, such as white spots of fungal hyphae growth, after just 1 or 2 days of shelf-life [39, 57, 58].

Despite there are many studies on postharvest use of MAP, to our knowledge, there are very few studies in literature regarding the influence of Argon and MAP on the shelf life and quality characteristics of small fruits. Particularly, the use of MAP in *Morus alba* fruit with Argon was not investigated yet. Stakeholders in the mulberry supply chain have raised concerns on the very short shelf life of fresh mulberry fruit sold in retail packages. In fact, because of high costs of production and wastes during postharvest storage, packaging, and transport, the mulberry supply chain is considered among the most unprofitable considering also high financial investments and labor required in the activities of the chain. For these reasons, in this study, several gas compositions were explored in order to ensure and maintain the quality characteristics of freshly harvested mulberries. Particularly, the objective of this research was to evaluate the effect of different MAP treatments (on quality parameters of mulberry fruit, stored at 4°C up to 12 d.

## 2. Materials and Methods

### 2.1. Plant Material

Fresh Italian white mulberry fruit (*Morus alba* L.) cv. ‘Kokuso 21' grown in Sicily (Southern Italy) in a commercial orchard located in Partinico (38°06′ N, 13°07′ E, 103 m a.s.l.) consisting of 13-year-old trees trained to a vase shape were harvested at commercial maturity stage in the first week of June 2019. The commercial maturity stage of mulberry fruit cv. 'Kokuso 21' is reached when the fruit shows a total soluble solid content of 13.0 (±0.4) °Brix [58]. Mulberry fruits that were over or under ripe and with slight injuries or defects were removed. After harvesting, the suitable fruit was immediately transferred by a refrigerated car within 2 hrs to the postharvest laboratory of the University of Palermo where it was processed within 3 hrs.

### 2.2. Experimental Design

To understand the effect of MAP treatments, the experiment was designed according to a full randomized block design with 4 main treatments: CTR1, CTR2, MAP1 and MAP2. Four storage times were tested: 0 (T_0_), 4 (T_4_), 8 (T_8_), and 12 (T_12_) days for each treatment and stored at 4 ± 1°C and 90 ± 5% RH.

Then, 2160 fruits were sampled as follows: 135 mulberry fruits per bag × 3 bags × 4 treatments × 4 times of storage were analyzed.

Therefore, the four treatments were as follows:

CTR1: 20.9% O_2_+0.04% CO_2_ (passive-MAP)

CTR2: 10% O_2_+5% CO_2_+85% N_2_

MAP1: 4% O_2_+6% CO_2_+90% N_2_

MAP2: 10% O_2_+5% CO_2_+85% Ar

Three hours after harvesting, the stem was cut to obtain uniform samples, and the fruits were irradiated for 30 minutes with ultraviolet germicidal light (UV-C; 180-280 nm with maximum at *λ* = 254 nm) to control microbial deterioration. Before packaging, fruits were washed with distilled water (5°C) and sanitized in 200 *μ*LL-1 Ox-Virin (solution of hydrogen peroxide and peroxyacetic acid; 0.5% *w*/*v*) for 2 minutes.

Subsequently, the fruits were placed in low-density polyethylene bags (LDPE, Orved, S.p.A., Musile di Piave, Venezia, Italy -90 *μ*-80 mm-500 cm^3^ films were the following: permeation to O_2_ (cm^3^ m^−2^ day^−1^): 4050; permeation to CO_2_ (cm^3^ m^−2^ day^−1^): 14000; OTR: 7500 cm^3^ m^−2^ day^−1^; WVTR: 6.5 g m^−2^ day^−1^under modified atmosphere packaging, using a digitally controlled packaging machine (VM 16 Orved S.p.A, Italy).

The relationship between the quantity of product and the gas mixture injected was 1 : 2 (*v*/*v*). Sensory and physicochemical analyses were carried out on three bags per treatment at each storage time to evaluate the shelf life of the fruit.

### 2.3. Weight Loss

The net weight loss of each bag was measured by a two-decimal precision digital scale (Gibertini EU-C 2002 RS, Novate Milanese, Italy), and the values were expressed as relative percentages of mean and standard deviation (1):

(1) $$\begin{array}{c} Weight\;loss\left(\%\right)=\left[\left(\text{Wi}-\text{Wd}\right)/\text{Wi}\right]\times 100, \end{array}$$

where Wi is the initial weight and Wd is the weight measured at the end of each storage time.

### 2.4. Juice Content

The pulp of fruit was extracted using a centrifugal juicer (Centrika Metal, Mod. 0173, Ariete, Italy), and the juice extracted (J) was expressed as mL of juice per 100 g of pulp.

### 2.5. Total Soluble Solid

Total Soluble Solid (TSS) content was determined using a pocket refractometer Atago Pal-1 (Atago Co., Ltd, Tokyo, Japan). At each storage time, twenty fruits per bag were taken and squeezed together to obtain one homogeneous juice sample per treatment which was used for repeated readings. The results were expressed as °Brix.

### 2.6. Titratable Acidity

The titratable acidity (TA), expressed as malic acid (g malic acidL^−1^), was determined by titration of 10 mL of juice with 0.1 M NaOH to an endpoint of pH 8.1, with a Crison Compact titrator pH-meter (Crison Instruments, SA, Barcelona, Spain).

### 2.7. Surface Color

Color was measured in terms of L^∗^, a^∗^, and b^∗^ values under CIELab Color System using a portable colorimeter (Minolta CR-400 Chronometer; Konica Minolta Sensing, Osaka, Japan), using 10 fruit for each treatments. Two readings were taken for each fruit, for a total of 20 readings per treatment.

Results, expressed as chroma (C∗) and hue angle (h°), were determined using a∗ and b∗ values according to Equations (2) and (3). The chroma value describes brightness, while the hue angle represents a coordinate in a standardised color space.

(2) $$\begin{array}{c} C^{*}=\left(a^{*}+b^{*2}\right)0.5 \end{array}$$

(3) $$\begin{array}{c} h^{{}^{\circ}}=\arctan\left(b^{*}/a^{*}\right) \end{array}$$

### 2.8. Headspace Gas Analysis

At each sampling date, two bags per treatment were used to measure the CO_2_ and O_2_ content in the headspace of the bag using a Dansensor Checkpoint O_2_ PBI analyzer and a CO_2_ analyzer (Topac, Hingham, MS, USA) equipped with zirconium and infrared detectors, respectively. Gas samples were taken from the bags with a 20 mL syringe. Results were expressed as the average O_2_ and CO_2_ % for the three readings for each bag.

### 2.9. Sensory Profile

Sensory evaluation test was performed by an evaluation team of 30 panelists (sixteen men and fourteen women aged between 25 and 60 years) with a good background and knowledge of this type of evaluation [59–61]. During the preliminary meetings, 14 descriptors were selected on the basis of citation frequency (>60%) for the definition of the sensory profile, which are the following: Skin color (PC), Consistency (CN), Mulberry odor (MO), Fruity odor (FO), Off-odor (OO), Acid (AC), Sweet (SW), Bitter (BT), Juiciness (JUI), Astringent (AST), Mulberry flavor (MF), Fruity flavor (FF), Off-flavor (OF), and Total evaluation (TE).

The evaluation was carried out from 10.00 a.m. to 12.00 p.m. in a proper room with individual cabins under white lights. The samples were taken from the cold room 1 hour before being tasted and were presented in a white plastic plate [62]. Each panelist received in random order a sample of 2 anonymous mulberry fruits in each plate, labeled with numbers. Sparkling water was provided for rinsing between each sample.

The judges evaluated the intensity of each descriptor by assigning a score, each score represented a different level of intensity of the quality descriptors. The panelists scored the descriptors according to a 9-point intensity scale: 1 = no sensation, 2 = barely recognizable, 3 = very weak, 4 = weak, 5 = light, 6 = moderate, 7 = intense, 8 = very intense, and 9 = extremely intense [62].

### 2.10. Statistical Analysis

A Two-Way ANOVA was performed to evaluate the effect of the cold storage period and MAP on quality parameters using the univariate general linear model procedure. Statistical analysis was carried out using the SISTAT 13.2 statistical software (Systat Software Inc., CA, USA). Significant differences (*p* ≤ 0.05) among treatments during each storage time and for each treatment over storage were evaluated by Tukey's multiple range test (Tukey HSD test). Pearson's correlation was also performed.

## 3. Results and Discussions

### 3.1. Physical Analyses

#### 3.1.1. Weight Loss

One of the characteristics of small fruits like mulberry or strawberry which contributes to their highly perishable character is the rapid water loss [63]. Modified atmosphere packaging reduces water loss by maintaining a relatively high humidity in the headspace atmosphere [64].

**Figure 1:**
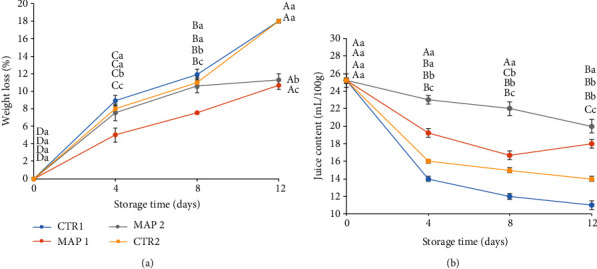
Pomological traits of mulberry fruit at 0, 4, 8, and 12 days of storage at 4 ± 1°C and 90 ± 5% RH (relative humidity) after all treatments. (a) Weight loss, %. (b) Juice content, mL/100 g. Means ± SD with different letters are significantly different at *p* ≤ 0.05 using Tukey's HSD test. Different lowercase letter denotes significant differences (*p* ≤ 0.05) among different treatments for the same sampling time. Letter “a” or “A” denotes the highest value. Different capital letters denote significant differences (*p* ≤ 0.05) among different sampling times for the same treatment.

In this study, all treatments showed a gradual loss of weight during storage due to transpiration (Figure 1(a)). However, significant differences were found (*p* ≤ 0.05) in net weight loss for different treatments, particularly from day 8 of storage (Figure 1), when fruits treated with MAP1 and MAP2 had a lower weight loss: 10% and 10.5%, compared to CTR1 and CTR2 which had a weight loss of 18.09% and 18%, respectively. According to literature [65], the lower weight loss of samples treated with MAP1 and MAP2 could be a consequence of lower activity of the enzymes responsible for softening of pericarp (pectinesterase, polygalacturonase, and beta-galactosidase) in fruits subjected to these treatments and for loss of cellular juice. In particular, for fruits treated with Ar (MAP2), the lower weight loss compared to CTR1 and CTR2 could be due to the fact that argon has a higher capacity to form clathrate hydrate than nitrogen [66]. Argon combines with water at an appropriate pressure to form clathrate hydrate, which limits the fluidity of the water and thus reduces water loss in fruits and vegetables [67, 68]. In fact, in general, the effectiveness of nonconventional gases has been suggested in relation to their ability to lower the water activity of packaged food [69]. Therefore, the results obtained have revealed that the treatment with Argon maintained the weight of the fruit, and this is in agreement with previous studies [70].

From the data obtained, we note that fruits stored in MAP1 also lose less weight than CTR fruits, in agreement with several authors who state that nitrogen is used to safely balance shelf life extension with optimal organoleptic properties of the product [71].

#### 3.1.2. Juice Content

Mulberry juice is a popular drink among consumers, because this fruit is considered healthy for its intrinsic characteristics [72]. Generally, consumers expect fruit to give the sensation of juiciness, and research on small fruits has shown that the juice content is influenced by several factors and not only related to water content [73].

In particular, as reported in the literature, mulberry juice is rich in biologically active compounds [74], and, therefore, keeping the mulberry fruit completely unaffected by any damage is very important from a nutritional and commercial point of view.

The decrease in juiciness is due to the depolymerization of pectins following the action of *β*-galactosidase and pectinesterases [75], which especially in fleshy fruits hydrolyze pectins to peptic acids and methyl alcohol, reducing their gelling power and making the pulp softer. In addition, Asrey et al. [76] studied the specific activities of polygalacturonase and pectinesterase which show higher levels at harvesting at commercial maturity, as in the case of the mulberry used in our experiment. At the eight days (T_8_), we note that CTR1, CTR2, and MAP1 have reached the lowest value, confirmed by a visual loss of the fruit structure. MAP2 fruits, on the other hand, maintain the most constant juiciness values; for this reason, the MAP2 treatment appeared the most appropriate. In all our treatments, the juice content decreases in the first 4 days, probably due to low-temperature storage (Figure 1(b)); specifically, CTR1 decreases significantly, while MAP and CTR2 treatments show a less marked trend.

Furthermore, the results obtained for Ar-treated fruit can be explained by referring to what was reported by Zhang et al. [77] who state that argon has a better ability than nitrogen to reduce the level of dissolved oxygen, the presence of which is necessary for tyrosinase to catalyse the reaction, suggesting that argon can inactivate certain chemically active sites on the enzyme more effectively than nitrogen. Furthermore, Zhang et al. [77] state that argon treatment has a slightly greater effect on malic dehydrogenase than nitrogen treatment. This may be due to the fact that the higher solubility of argon compared to nitrogen may generate a higher affinity for the enzyme's inhibitory site.

Finally, argon appears to be a gaseous inhibitor for enzymes related to browning and respiration and therefore could play an important role in maintaining the quality of fresh fruit and vegetables to replace some chemical treatments that cause potential health risks.

The water solubility of Ar and N_2_ is 0.034 and 0.016 g L^−1^ [78], respectively, so the greater capacity of Ar to delay the physiology of the mulberry compared to N_2_ could be due to the greater capacity of these gases to dissolve in the aqueous layer of the fruit and then through the pulp cells. Therefore, argon may have inactivated certain chemically active sites on enzymes more effectively than N_2_ by reducing the level of dissolved oxygen, the presence of which is necessary for oxidative enzymes to catalyse metabolic reactions.

#### 3.1.3. Total Soluble Content and Titratable Acidity

Total soluble solids are a critical factor in determining fruit quality and consumer acceptability. Sugars are the main soluble metabolites and include glucose, fructose, and sucrose, accounting for 99% of the total sugar content [79]. TSS which mainly includes sugars and acids is closely related to taste and indicates the degree of maturity [80].

**Figure 2:**
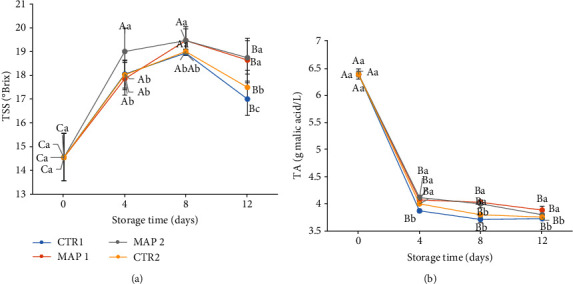
Pomological traits of mulberry fruit at 0, 4, 8, and 12 days of storage at 4 ± 1°C and 90 ± 5% RH (relative humidity) after all treatments. (a) TSS, °Brix. (b) TA, g malic acid/L. Means ± SD with different letters are significantly different at *p* ≤ 0.05 using Tukey's HSD test. Different capital letter denotes significant differences (*p* ≤ 0.05) among different treatments for the same sampling time. Different lowercase letter denotes significant differences (*p* ≤ 0.05) among different treatments for the same sampling time. Letter “a” or “A” denotes the highest value. Different capital letters denote significant differences (*p* ≤ 0.05) among different sampling times for the same treatment.

At harvest time, the values of TSS and TA were 14.55°Brix and 6.39 g malic acid/L, respectively. Observing the evolution of TSS and TA of mulberry, as expected, that after 4 days of cold storage, TSS increased and TA slightly decreased. TSS of our samples generally increased during the first 8 days of cold storage (24% CTR1, 25% CTR2, 27% MAP1, and 30% MAP2) and then decreased (Figure 2(a)). The TSS values decreased in the period from T_8_ to T_12_ more for CTR1 and CTR2 fruits than for MAP1 and MAP2 fruits. This is due to the physiological ripening processes that determine the accumulation of sugars used as substrate in breathing processes [81] but is less pronounced in MAP-treated fruit due to the use of gas.

**Figure 3:**
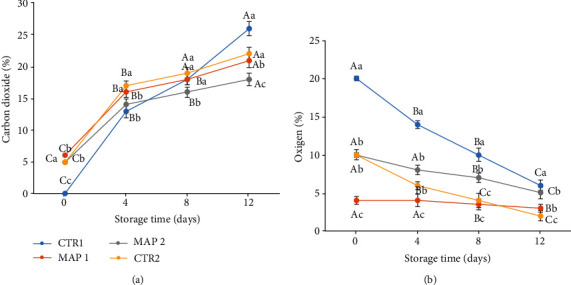
(a) Carbon dioxide content % (CO_2_) and (b) oxygen % (O_2_) inside packages with mulberry fruit stored at 4 ± 1°C and 90 ± 5% RH (relative humidity). Means ± SD with different letters are significantly different at *p* ≤ 0.05 using Tukey's HSD test. Different capital letter denotes significant differences (*p* ≤ 0.05) among different treatments for the same sampling time. Different lowercase letter denotes significant differences (*p* ≤ 0.05) among different sampling times for the same treatment. Letter “a” or “A” denotes the highest value.

Fruits, due to the use of N_2_ and Ar gases, consumed less O_2_ (no hypoxic conditions were reached), as can be seen from Figure 3(a). Therefore, since we know that ripening is inversely proportional to the rate of respiration of the fruits, it is believed that the gases decreased the rate of respiration and therefore the consumption of organic substrates [82].

On the other hand, in agreement with the literature [39], we know that nitrogen is also used to replace oxygen in MAP products to prevent rancidity and inhibit the growth of aerobic organisms, and, on the other hand, Ar has some chemically active sites on enzymes and interferes in the evolution of soluble solids content during the ripening process. Rodriguez and Zoffoli [83] showed that in guava fruit, the significant increase in total sugars observed after the climacteric peak can be due to the increased activity of the enzymes responsible for the formation of complex sugars.

Moreover, as we can see from the data, the TSS values in mulberries preserved in MAP1 and MAP2 follow a more linear development than in fruit with CTR1 and CTR2 treatments.

In particular, fruits treated with MAP2 keep TSS content higher, until T_8_, than other treatments, confirming that Ar interferes in the evolution of soluble solids [39].

The content of acid (TA), mostly malic acid, has gradually decreased over the storage period; as a result, the quality of the mulberries has decreased (Figure 2(b)). Before us, a similar behavior was observed by Caner [84], who attributes this to the dissolution of CO_2_ in the water present on the surface of the fruit, generating carbonic acid and acidifying the fruit. The decrease in acidity was depending on the storage time (*p* < 0.05); this shows a high decrease in the values detected in all treatments during the first 4 days of storage and then stabilized at constant and established values up to 12 days of storage.

The sugar/acid ratio was evaluated, which is considered an index of fruit quality for fruit [85], and it is important to note that fruits treated with MAP show an increasing sugar/acidity ratio during the storage period (2.28-4.40; 2.28-4.50; 2.28-5.45; 2.28-4.96 for CTR1; CTR2; MAP1; MAP2, respectively), thus suggesting a good quality characteristics for consumption even after storage [86].

### 3.2. Surface Color

Color is an important sensory characteristic of mulberry which affects the identification and recognition of the degree of maturity of the fruit and of the product quality; therefore, minimizing pigment losses during processing is one primary concern of the processor [45].

Maintaining the intrinsic color of fresh fruit is often used as a quality indicator and has a substantial impact on consumer acceptance [87]. Color development occurs as fruit matures, which is mainly due to the synthesis and degradation of anthocyanin, a pigment that contributes to the red color [88].

During the storage period, MAP1 and MAP2 were better at preserving luminosity, while CTR1 and CTR2 fruits became significantly darker with the lowest L∗ value (data not shown).

**Figure 4:**
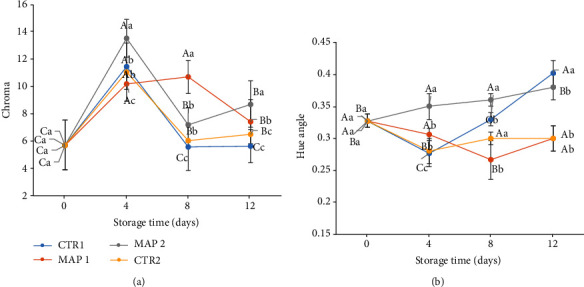
Color characteristics of mulberry fruit at 0, 4, 8, and 12 days of storage at 4 ± 1°C and 90 ± 5% RH (relative humidity) after all treatments. (a) Chroma value. (b) Hue angle. Means ± SD with different letters are significantly different at *p* ≤ 0.05 using Tukey's HSD test. Different lowercase letter denotes significant differences (*p* ≤ 0.05) among different treatments for the same sampling time. Letter “a” or “A” denotes the highest value. Different capital letters denote significant differences (*p* ≤ 0.05) among different sampling times for the same treatment. Letter “a” denotes the highest value.

Chroma values increased initially for all samples. However, since time T_8_, they decreased, particularly the CTR1 samples, which were therefore less bright (lower chroma value). Moreover, as we can see from the data (Figures 4(a) and 4(b)), the highest chroma values were reported in both MAP treatments, probably due to the gas mixture that slowed down the oxidative processes [89], and this supports the observation that MAP preserved the color of mulberry fruit.

In the presence of oxygen, the fruit would suffer an enzymatic browning reaction [90, 91], which is undesirable as color is one of their most important parameters affecting consumer acceptance and purchase. Under normal conditions, in fruit, substrates and enzymes are distributed in different cell regions and enzymes do not promote browning. However, under adverse conditions such as senescence, the active oxygen metabolites are unbalanced, leading to lipidification of the cell membrane, destruction of the membrane structure, degradation of the cell structure, and the promotion of a large amount of contact between substrates and enzymes which leads to browning of the flesh and finally to loss of economic and nutritional value [92]. The oxidative lesion of the membrane, in fact, allows the mixing of the normally separated enzyme (PPO) and oxidisable substrates (polyphenols) [54].

### 3.3. Headspace Gas Analysis

The atmosphere in the packages depends on the initial gas added, the permeability of the packages, and the respiration of the fruit which produces CO_2_ and consumes O_2_ [93, 94]. Therefore, the composition of the gas is always in a state of dynamic change, and the concentration of O_2_ decreases and CO_2_ increases. The levels of O_2_ and CO_2_ detected in the headspace of the sample package during storage are shown in Figures 3(a) and 3(b). The initial gas composition changed rapidly for all treatments, and, as expected, we observe a decrease in O_2_ concentration in the headspace, as well as an increase in CO_2_ concentration during storage.

The final CO_2_ content was 17% for MAP2 and 21% for MAP1, while it was 26% for CTR1 (passive-MAP) samples (Figure 3(a)). The changes observed can be attributed not only to fruit respiration but also to gas permeability through the LDPE film, as already reported by Hodges [95]. In general, the concentration of O_2_ decreased rapidly during the first days of storage.

In particular, with regard to MAP1, the increase in CO_2_ levels during storage caused a decrease in respiration and therefore in O_2_ utilisation by the fruit [82].

With regard to CTR1, what we noticed was that the fruits consumed more than half of the initial O_2_ concentration (Figure 3(b)) and simultaneously produced much more CO_2_ than in the other treatments. This higher rate of respiration resulted in damage to the fruit which led to a greater loss of weight as they became very dehydrated and deliquescent.

In the MAP2 samples, CO_2_ increased progressively during the storage period and at T_12_ was lower than in the other treatments. Similarly, the O_2_ concentration decreased slightly from T_8_, and this suggests that no undesirable changes occurred in the fruit, including the development of off-flavors [96], as also confirmed by the sensory analyses.

### 3.4. Sensory Profile

**Figure 5:**
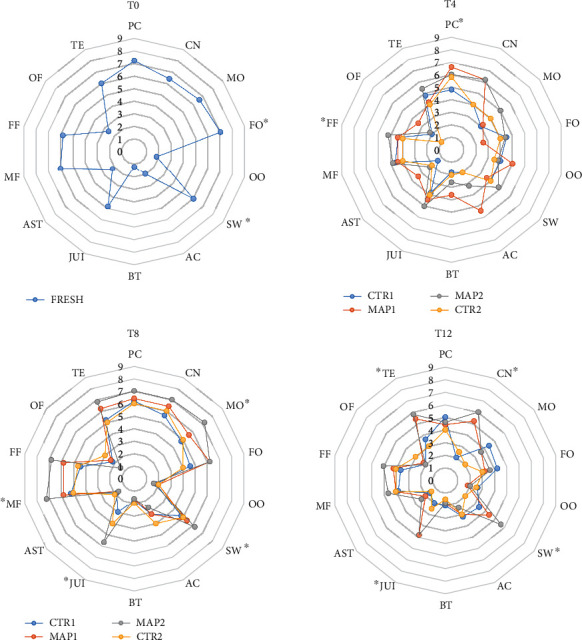
Sensory analyses of treated and untreated mulberry fruit at 0, 4, 8, and 12 (T0, T4, T8, T12) days of storage at 4° ± 1°C and 90% ± 5% RH. Descriptors legend: Peel color (PC), Consistency (CN), Mulberry odor (MO), Fruity odor (FO), Off-odor (OO), Sweet (SW), Acid (AC), Bitter (BT), Juiciness (JUI), Astringent (AST), Mulberry flavor (MF), Fruity flavor (FF), Off-flavor (OF), and Total evaluation (TE). For each descriptor, the values marked with ^∗^ indicate significant differences between treatments. Data are the mean of 60 replications from one replicate of 60 fruit each.

The fresh fruit has been much appreciated by panelists and specifically immediately after harvest (T_0_) because of its intense flavor, aroma, and uniform epicarp color. After 4 days, it was possible to observe significant differences between all treatments. The highest score for the descriptors CN, PC, TE, and FF were given to fruit treated with MAP2, which maintained the values of the descriptors of fruit with almost no quality damages, followed by fruit treated with MAP1. In CTR fruit, it is possible to see a decrease of the organoleptic characteristics during the storage period. In general, in CTR, there was a decrease in the smell of mulberry with the appearance of off-flavor, probably due to the activation of fermentation processes. The fruit treated with MAP2 maintained high values of juiciness, sweetness, and consistency, which are very important parameters commercially. After 8 days (T_8_), significant differences between CTR-, MAP1-, and MAP2-treated fruit begin to appear. CTR fruit had a general decrease of all descriptors, due to the normal physiological decay of the fruit in a passive atmosphere [97]. Fruit treated with MAP1 maintained the organoleptic characteristics, and those treated with MAP2 had the highest values for the attractiveness of the fruit, demonstrating the effectiveness of this gaseous mixture in maintaining the quality of the fruit. In fact, these fruits had very good values of consistency color, sweetness, and odor. At T_12_, the descriptor that showed a significant decrease was the color. The fruit was opaque and with superficial deliquescence. The most appreciated fruits were those treated with MAP2 and the sensory analysis confirmed these results, highlighting that the MAP treatment with Argon MAP2 maintained all the organoleptic characteristics in terms of sweetness, flavor, and juiciness, providing valuable support in promoting the use of this treatment for prolonging the shelf-life of a fruit as delicate as these ones (Figure 5).

## 4. Conclusions

Modified atmosphere packaging (MAP) and argon treatments were found to be optimal storage treatments for mulberry fruit, maintaining their TSS but also their color, thus extending their shelf-life during refrigerated storage by up to 12 days. The results obtained show that the MAP1 (4% O_2_+6% CO_2_+90% N_2_) and MAP2 (10% O_2_+5% CO_2_+85% Ar) treatments, combined with storage at low temperatures (4 ± 1°C), allowed to have fruit with good chemical-physical and organoleptic characteristics during 12 days. After 8 days of storage, MAP2 treatment showed optimal results in maintaining the juiciness, color, and TSS/TA ratio compared to fruit treated with MAP1 and CTR1 or CTR2. In particular, when comparing CTR2 and MAP2, it is possible to observe the positive effects of argon for the storage weight (over 80% for CTR2), the solid soluble content, the color, and the lower percentage of CO2 inside the bag.

It should be noted that although MAP with Ar prolongs the shelf life of the fruit after harvesting, the costs of the application are not low, and industrial use is useful only if the final product comes out with a high price on the market, but this is the case of mulberry fruit. Nevertheless, these findings clearly highlight that the use of Ar in the gaseous mixture, and the storage at low temperature provide a longer shelf life to this fruit of high intrinsic quality. Therefore, these findings may have useful implications for producers, stakeholders, and researchers, because a collaboration to allow the entrepreneurs of the mulberry supply chain to apply this technique will contribute to enhance the commercialization of Italian mulberry fruit in the foreign markets.

## Data Availability

The data used to support the findings of this study are included within the article.
